# Role of Glycosylation/Deglycolysation Processes in *Francisella tularensis* Pathogenesis

**DOI:** 10.3389/fcimb.2017.00071

**Published:** 2017-03-21

**Authors:** Monique Barel, Alain Charbit

**Affiliations:** ^1^Sorbonne Paris Cité, Bâtiment Leriche, Université Paris DescartesParis, France; ^2^Institut National de la Santé et de la Recherche Médicale, Institut Necker-Enfants Malades, INSERM U1151 -Team 11, Pathogenesis of Systemic InfectionsParis, France; ^3^Centre National de la Recherche Scientifique, UMR8253Paris, France

**Keywords:** glycosylation, host-pathogen interaction

## Abstract

*Francisella tularensis* is able to invade, survive and replicate inside a variety of cell types. However, *in vivo F. tularensis* preferentially enters host macrophages where it rapidly escapes to the cytosol to avoid phagosomal stresses and to multiply to high numbers. We previously showed that human monocyte infection by *F. tularensis* LVS triggered deglycosylation of the glutamine transporter SLC1A5. However, this deglycosylation, specifically induced by *Francisella* infection, was not restricted to SLC1A5, suggesting that host protein deglycosylation processes in general might contribute to intracellular bacterial adaptation. Indeed, we later found that *Francisella* infection modulated the transcription of numerous glycosidase and glycosyltransferase genes in human macrophages and analysis of cell extracts revealed an important increase of N and O-protein glycosylation. In eukaryotic cells, glycosylation has significant effects on protein folding, conformation, distribution, stability, and activity and dysfunction of protein glycosylation may lead to development of diseases like cancer and pathogenesis of infectious diseases. Pathogenic bacteria have also evolved dedicated glycosylation machineries and have notably been shown to use these glycoconjugates as ligands to specifically interact with the host. In this review, we will focus on *Francisella* and summarize our current understanding of the importance of these post-translational modifications on its intracellular niche adaptation.

## Introduction

Protein glycosylation is one of the most common post-translational modifications (PTM) of proteins, as present in all kingdoms of life. It consists in the covalent attachment of glycans onto amino acid side chains, this reaction being catalyzed by an enzyme. In eukaryotic cells, glycosylation has significant effects on protein folding, conformation, distribution, stability, and activity. Particularly, the sugar chains of glycoproteins are essential for maintaining the order of intercellular interactions among all differentiated cells in multicellular organisms. Therefore, alterations in the sugar chains may range from being essentially undetectable to a complete loss in particular functions (Varki, 1993). Indeed, dysfunction of protein glycosylation may lead to development of diseases like cancer and pathogenesis of infectious diseases (Moran et al., 2011). In the innate immune system, which is the major actor for protection against microbial pathogens, several host glycoproteins have been shown to function as pattern recognition receptors (PRRs), involved in pathogen binding (Di Gioia and Zanoni, 2015). Cell-surface glycoproteins facing the extracellular environment are ideally located to facilitate this host–pathogen interaction. The receptors of the innate immune response i.e., Toll-like receptors (TLRs) and nucleotide oligomerization domain (NOD-like) receptors (NLRs) are glycoproteins. In the adaptative immune response, the major components, which include class I and class II major histocompatibility complex proteins, chemokine and cytokine receptors, and essentially all cytokines and chemokines are glycosylated (Opdenakker et al., 2016).

Bacterial pathogens have also evolved dedicated glycosylation machineries. When compared to higher organisms, bacteria are capable of producing an extraordinary amount of unique and diverse glycans, which are principally attached to the cell surface, and secreted molecules. Bacteria are able to use these glycoconjugates as a range of unique and specific ligands, which specifically interact with the host (Tytgat and de Vos, 2016). Bacteria are covered with various types of carbohydrate moieties. These surface-exposed bacterial structures are often called pathogen-associated molecular patterns (or PAMPS).

Oligosaccharides may either mediate “specific recognition” events or provide “modulation” of biological processes. For example, they may allow interaction of bacterial proteins with host-derived proteins or they may modulate bacteria- and/or host-related events (Bastos et al., 2017). All these events may be essential for bacterial colonization, its survival and the subsequent infection. Therefore, host immunization may be dependent on these PTM, whether mediated by the pathogen or by the host.

*Francisella tularensis* is a Gram-negative bacterium causing the zoonotic disease tularemia in a number of mammalian species, including humans (Sjöstedt, 2011). *F. tularensis* invades, survives and replicates inside a variety of cell types, including phagocytic and non-phagocytic cells of various species (Meibom and Charbit, 2010), as well as arthropod-derived cells (Santic et al., 2010). “*In vivo*,” *F. tularensis* preferentially enters host macrophages (Clemens et al., 2005), rapidly escapes to the cytosol where it actively multiplies (Case et al., 2014). While the cytoplasm was initially considered as a safe nutrient-replete haven (Ray et al., 2009), it is now clearly established that the host cytosol may be a harsh environment by depriving nutrients against invading bacteria (Abu Kwaik and Bumann, 2013; Zhang and Rubin, 2013). Conversely, invading intracellular pathogens may also “steal” nutrients of the host cell that, in turn, needs to adapt its metabolism to control its cytosolic content (Barel et al., 2015). Indeed, upon addition of gluconeogenic substrates, such as oaxaloacetate and pyruvate, to the cell culture medium increased intracellular multiplication of *F. tularensis* LVS was observed, suggesting that these nutrients served as sources of glucose to feed multiplying bacteria.

We will herein summarize what is known about the glycosylation-deglycosylation processes occurring during *Francisella* infection, as observed from either the host or the pathogen.

## Host point of view

*Francisella* infection modifies numerous “glyco-genes” involved in glycosylation pathways in human macrophages. Indeed, using a glycan processing gene microarray (Chacko et al., 2011), we observed significant changes in the level of glycosyltransferase and glycosidase gene expression profiles in human THP-1 monocytes, infected for 24 h with *F. tularensis* LVS (Barel et al., 2016). Expression of eight genes, encoding four glycosyltransferases and four glycosidases, was down-regulated upon infection. These four glycosidase belonged to the EDEM family, which is involved in ER-associated degradation (ERAD). The expression of six genes was up-regulated upon infection, corresponding to five glycosyltransferases and one glycosidase. The up-regulated glycosyltransferases were involved either in *N*-glycosylation or in *O*-glycosylation of glycoproteins. The glycosidase gene whose expression was up-regulated, encoded the glycosidase HEXA, which is involved in the Hexosamine Biosynthetic Pathway (HBP) (Vaidyanathan et al., 2014).

Glycosylation occurred as soon as 1 h after entry of the bacteria into the cells. Only three proteins were found and characterized as carrying potential *N*-glycosylation residues, while nine proteins contained potential *O*-glycosylation residues. Among them, we characterized BiP/GRP78/HSPA5 protein, a member of the HSP70 heat shock protein family. BiP expression was increased both at transcription and translation level, by *F. tularensis* LVS infection immediately after binding to the cells. BiP glycosylation was also induced at early stage of infection. BiP being a key regulator of the UPR (Ni et al., 2009; Pfaffenbach and Lee, 2011), we hypothesized that the glycosylation-deglycosylation processes could be modified by *Francisella*. This could result in direct triggering of the UPR (including BiP) in infected cells with a decrease of the load of newly synthesized “abnormal” proteins. In addition, among the nine proteins containing potential O-glycosylation residues and being glycosylated by *Francisella* infection, we also found PRKCSH, the beta-subunit of glucosidase 2. This enzyme is acting upstream BiP, in the calnexin pathway, which is also involved in correcting misfolded proteins (Hetz et al., 2011).

Infection of human monocytes by *F. tularensis* LVS also triggered the deglycosylation of the glycosylated amino acid transporter SLC1A5 and other glycoproteins (Barel et al., 2012). Deglycosylation induced by *F. tularensis* LVS was maximum at 24 h when intracellular multiplication occurred and depended on the capacity of the bacteria to escape from the phagosomes (Barel et al., 2012). It was not an inhibition of glycosylation since tunicamycine had no inhibiting effect on this deglycosylation.

The enzymes involved in these glycosylation-deglycosylation mechanisms are still not characterized.

**Figure 1:**
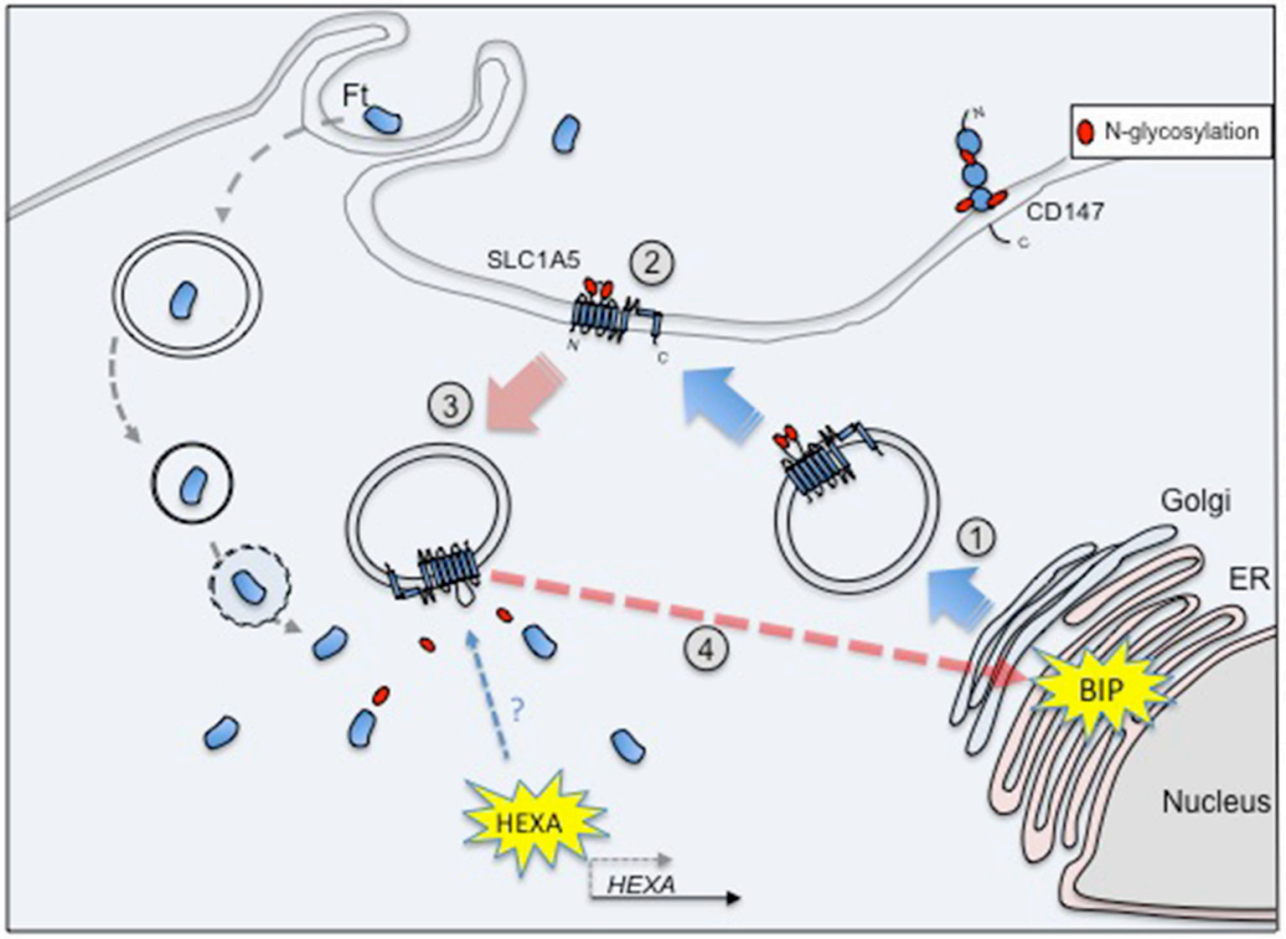
**SLC1A5 is glycosylated after passage into the Golgi ① and is exported to the membrane ②**. Its endocytosis into the cytoplasm renders it available to glucosidases ➂, e.g., HEXA, whose transcription level is increased upon *Francisella* infection. The deglycosylated form of SLC1A5 has been indeed localized only in the cytoplasm. In turn, these deglycosylated (and possibly misfolded) proteins could trigger the increase of BiP expression and its glycosylation ➃.

We tried to summarize the cascade of events triggered upon infection of macrophages by *Francisella* in the hypothetical model depicted in Figure 1. The transporter SLC1A5 was chosen as a prototypic glycosylated membrane protein. After its synthesis and translocation into the ER, the protein is transported to the Golgi where it is first glycosylated ➀ and, from there, addressed to the membrane via secretory vesicles. In the plasma membrane, SLC1A5 is present only as a glycosylated protein ➁ (Console et al., 2015). Upon re-entry into the cytoplasm via endocytosis, glycosylated SLC1A5 becomes available to glucosidases ➂ such as HEXA (whose expression is induced upon *Francisella* infection). The deglycosylated form of SLC1A5 has been indeed localized only in the cytoplasm (Console et al., 2015). This deglycosylated form of the protein (possibly misfolded) could trigger increase of BiP expression and its glycosylation ➃.

It is tempting to suggest that the intracellular survival of *Francisella* would be favored both by the control exerted on the UPR response of the host and by the availability of free oligosaccharides resulting from deglycosylation processes, that could serve as nutrients.

## Pathogen point of view

A large number of bacterial proteins have been found to be glycosylated (Tan et al., 2015). They show a surprising degree of diversity, both within and between bacterial species. Protein glycosylation can be classified according to the glycosidic linkage. Attachment to the amide nitrogen of asparagine (Asn) is known as *N-*glycosylation, with that of serine or threonine (Ser/Thr) to the hydroxyl oxygen being known as *O-*glycosylation. *N-* and *O-*linked glycosylation may occur either through the action of an oligosaccharyltransferase (OST) or via the action of glycosyltransferases (GTs). OSTs substrates are lipid-linked oligosaccharides while the GTs substrates are usually nucleotide-activated sugars. It was only very recently (Dankova et al., 2016) that the glycosylation machinery of *Francisella* was found to involve a variety of sugar biogenesis enzymes, glycosyltransferases, a flippase, and a protein-targeting oligosaccharyltransferase. As both type A and type B strains of *F. tularensis* subspecies expressed an *O*-linked protein glycosylation system, which utilizes core biosynthetic and assembly pathways, *O*-linked protein glycosylation may be a feature common to members of the *Francisella* genus (Egge-Jacobsen et al., 2011).

The initial attempts to elucidate the glycan repertoire of *Francisella* and their structures had failed because of the enzymatic and chemical release techniques used. Some proteins were found after transcriptional profiling of mutants. Indeed, FTT_0905 was characterized as a glycosylated Type IV pili protein, which is transcriptionally regulated by MglA. As MglA controls the expression of the *Francisella* pathogenicity island, FTT_0905 was considered as a new virulence factor (Brotcke et al., 2006). However, by mapping the glycoproteome of the FSC200 strain of *F. tularensis* subsp. *holarctica*, several candidate proteins were found that could be target for glycosylation as DsbA (FTH_1071), an uncharacterized protein FTH_0069, FopA, Tul4, and LemA (Balonova et al., 2010). In contrast, the PglA protein was identified as a targeting oligosaccharyltransferase because it is necessary for PilA glycosylation in *F. tularensis* (Egge-Jacobsen et al., 2011). Indeed, this protein undergoes multisite O-linked glycosylation, with a pentasaccharide of the structure HexNac-Hex-Hex-HexNac-HexNac. PglA is highly conserved in *Francisell*a genus, supporting the general feature of *O*-glycosylation. Then, the detailed characterization of the DsbA glycan and the putative role of the FTT0789–FTT0800 gene cluster in glycan biosynthesis were reported (Thomas et al., 2011). Indeed, these authors observed that the essential virulence factor DsbA migrated as multiple protein spots on two-dimensional electrophoresis gels. The protein was modified with a 1,156-Da glycan moiety in O-linkage. The glycan is a hexasaccharide, comprised of N-acetylhexosamines, hexoses, and an unknown monosaccharide. Loss of DsbA glycan modification was obtained by disruption of two genes within the *FTT0789*–*FTT0800* putative polysaccharide locus, including a *galE* homolog (*FTT0791*) and one gene encoding a putative glycosyltransferase (*FTT0798*). As the mutants remained virulent in the murine model of subcutaneous tularemia, it indicated that glycosylation of DsbA does not play a major role in virulence under these conditions (Thomas et al., 2011). When defining the previously uncharacterized FTH_0069 protein as a novel glycosylated lipoprotein required for virulence, Balonova et al. (2012) also showed that the glycan structure modifying its two C-terminal peptides was identical to that of DsbA glycoprotein, as well as to one of the multiple glycan structures modifying the type IV pilin PilA. They therefore suggested a common biosynthetic pathway for the protein modification and a relationship between synthesis of the *O*-antigen and the glycan in the early steps of their biosynthetic pathways. Indeed, the *pglA* gene, encoding pilin oligosaccharyl transferase PglA, was involved in both pilin and general *F. tularensis* protein glycosylation.

In another study on activation of pulmonary inflammation after *F. tularensis* Schu S4 exposure (Walters et al., 2013), altered expression level of bacteria-specific mRNA transcripts was found. Among these transcripts, a hypothetical protein FTT_0797 was characterized which shared homology with a glycosyl transferase. This protein is part of a gene cluster, which is thought to encode a polysaccharide additional to the lipopolysaccharide O antigen. Another protein, encoded by *FTS_1402*, was found to be involved in glycoprotein synthesis and to also contribute in part to LPS/capsule and/or Capsule Like Complex (CLC) production (Dankova et al., 2016). The resulting *FTS_1402* mutant presented more sensitivity to serum complement.

All these proteins are summarized in Table 1.

**Table 1:** **Genes published involved in glycosylation pathway**.

**Published Gene**	**Gene Number (FTT)**	**Protein name**	**Characteristics**	**Function**	**References**
FTT_0905		Type IV pili glycosylation protein	Glycosylated Type IV pilus	Virulence Factor	Brotcke et al., 2006
FTH_1071	Dsba	DsbA	Glycan Biosynthesis	Virulence Factor not affected when glycan is lost.	Thomas et al., 2011
FTH_0069			Putative Glycosylation		Balonova et al., 2010
fopA		FopA	Putative Glycosylation		Balonova et al., 2010
tul4		Tul4	Putative Glycosylation		Balonova et al., 2010
lemA		Lema	Putative Glycosylation		Balonova et al., 2010
pglA		PglA	Oligosaccharyltransferase	Pilin and Protein glycosylation	Egge-Jacobsen et al., 2011
FTT_0789		Ribulose-phosphate 3-epimerase	Glycan Biosynthesis		Thomas et al., 2011
FTT_0798		Glycosyl transferase family protein	Putative glycosyltransferase		Thomas et al., 2011
FTH_0069	FTT_1676	Hypothetical protein	Glycosylated lipoprotein	Virulence Factor	Balonova et al., 2012
FTT_0797		Glycosyl transferase family protein	Glycosyltransferase	Involved in O antigen glycosylation	Walters et al., 2013
FTS_1402	FTT_0793	ABC transporter	Putative glycan flippase	Involved in LPS and CLC product	Dankova et al., 2016

*FTT, Francisella tularensis ssp. tularensis; FTH, Francisella tularensis ssp. holartica; FTS, Francisella tularensis ssp. tularensis, FSC200 stain nomenclature*.

Concerning enzymes involved in degradation pathways, analysis of *F. tularensis* genomes showed a difference in the number of genes coding for proteins with such enzymatic activity (Table 2). Five genes were found in LVS, while only two genes were found in SchuS4 strain and only one gene in FSC200 strain. None of them was characterized.

**Table 2:** **Genes found in KEGG, with a putative deglycosylation function**.

***Francisella tularensis***	**Gene number**	**Name**	**Function**
*Subsp. tularensis SCHU S4*	FTT_0928c	Beta-N-acetylhexosaminidase [EC:3.2.1.52]	Beta-glucosidase
	FTT_0412c	Pullulanase [EC:3.2.1.41]	PulB; pullulonase
*Subsp. holarctica LVS (Live Vaccine Strain)*	FTL_1282	Beta-N-acetylhexosaminidase [EC:3.2.1.52]	Beta-glucosidase-related glycosidase
	FTL_1052		Putative glycosidase
	FTL_0482	Pullulanase [EC:3.2.1.41]	Pullulonase
	AW21_68	Glycosyl hydrolase family 3 N terminal domain	Hypothetical protein
	AW21_1415	Glycosyl hydrolase family 3 N terminal domain	Hypothetical protein
*subsp. holarctica FSC200*	FTS_1254	Beta-N-acetylhexosaminidase [EC:3.2.1.52]	Glycosyl hydrolase family protein
*Subsp. novicida U112*	FTN_0911	Alpha-glucosidase [EC:3.2.1.20]	Glycosyl hydrolases family 31 protein
	FTN_0627	chitinase [EC:3.2.1.14]	Chitinase, glycosyl hydrolase family 18
	FTN_0806	Beta-N-acetylhexosaminidase [EC:3.2.1.52]	Glycosyl hydrolase family 3
	FTN_1474	bglX	Glycosyl hydrolase family 3

## Role of post-translational modifications (PTM) on bacteria/host cell proteins

While two-third of all eukaryotic proteins are estimated to be glycosylated, the number of prokaryotic glycoproteins is still way behind understanding. This is mainly due to the enormous variability of their glycan structures and variations in the underlying glycosylation processes. In 2016, Schäffer and and Messner (2016) combined glycan structural information with bioinformatic, genetic, biochemical and enzymatic data for in-depth analyses of glycosylation processes in prokaryotes. This study included the major classes of prokaryotic (i.e., bacterial and archaeal) glycoconjugates without any example on *Francisella*. Furthermore, in a very recent publication (Bastos et al., 2017), while *F. tularensis* was shown to exhibit the largest number of glycoproteins in common with *M. tuberculosis* (*Mtb*), by sharing 16% of its glycoproteome, none of the glycosylated proteins of *Francisella*, as well as none of the enzymes involved in glycosylation pathway, have been found to play a specific role in pathogenesis. At the opposite, in *M. tuberculosis*, glycosylation of HbN, a truncated hemoglobin protein, was demonstrated to be necessary for its maintainance at the bacterial membrane and wall (Arya et al., 2013). Mutation in its mannose glycan linkage disrupted the facilitation of *Mtb* and *M. smegmatis* entry within the macrophages. These data suggested that glycosylation processes allowed *Mtb* survival within the hazardous environment of macrophages and the establishment of long term persistent infection in the host (Dey and Bishai, 2014).

Of note, *Francisella* did not belong to the list of prokaryotes that catalyzed glycosylation of host cell proteins (Bastos et al., 2017). In contrast, *Legionella* was cited as targeting eEF1A through effect of the glucosyl transferase Lgt1, with as result, the killing of eukaryotic cells (Belyi et al., 2008).

## Conclusion

While 146 examples of protein glycosylation were cited for *Francisella* and only 111 for *Helicobacter* pylori (Bastos et al., 2017), the importance of these PTM, observed in *Francisella* and those induced in the host, is still largely unknown, notably on the outcome of the infectious cycle. Indeed, a large correlation between glycosylation and bacterial pathogenicity has already been proven for various species e.g., *Campylobacter jejuni, Legionella* and enteropathogenic *Escherichia coli* (EPEC) (Lu et al., 2015).

*Francisella* infection modifies the unfolded protein response (UPR) (Barel et al., 2016) and manipulates autophagy (Miller and Celli, 2016). Both processes are involved in maintaining cellular homeostasis and helping destroy invading microorganisms. Glycosylation and deglycosylation could be involved in molecular mimicry of common host cell glycans therefore helping the bacteria to avoid immune recognition. At this stage, we have all the reasons to believe that the glycosylation-deglycosylation processes observed in THP-1 cells were originated from eukaryotic enzymes. However, we cannot formerly exclude that *Francisella* enzymes might also be involved. Glycans and glycan-binding receptors influence all stages of infection, starting from initial colonization of host epithelial surfaces to spreading in tissue and inducing inflammation or host-cell injury, which may results in clinical symptoms (Nizet and Esko, 2009). Therefore, knowledge of glycosylation pathways involved during *Francisella* infection remains fundamental for prevention and treatment strategies.

## Author contributions

MB and AC wrote the review.

### Conflict of interest statement

The authors declare that the research was conducted in the absence of any commercial or financial relationships that could be construed as a potential conflict of interest.

## References

[B1] Abu Kwaik Y. Bumann D. (2013). Microbial quest for food *in vivo*: “Nutritional virulence” as an emerging paradigm. Cell. Microbiol. 15, 882–890. 10.1111/cmi.12138 23490329

[B2] Arya S. Sethi D. Singh S. Hade M. D. Singh V. Raju P.. (2013). Truncated hemoglobin, hbn, is post-translationally modified in *Mycobacterium tuberculosis* and modulates host-pathogen interactions during intracellular infection. J. Biol. Chem. 288, 29987–29999. 10.1074/jbc.M113.507301 23983123 PMC3795296

[B3] Balonova L. Hernychova L. Mann B. F. Link M. Bilkova Z. Novotny M. V.. (2010). A multimethodological approach to identification of glycoproteins from the proteome of *Francisella tularensis*, an intracellular microorganism. J. Proteome Res. 9, 1995–2005. 10.1021/pr9011602 20175567 PMC3025813

[B4] Balonova L. Mann B. F. Cerveny L. Alley W. R. Chovancova E. Forslund A.-L.. (2012). Characterization of protein glycosylation in *Francisella tularensis* subsp. *holarctica*: identification of a novel glycosylated lipoprotein required for virulence. Mol. Cell Proteomics 11:M111.015016. 10.1074/mcp.M111.015016 22361235 PMC3394949

[B5] Barel M. Grall N. Charbit A. (2015). Pathogenesis of Francisella tularensis in Humans. Hoboken, NJ: John Wiley & Sons, Inc.

[B6] Barel M. Harduin-Lepers A. Portier L. Slomianny M.-C. Charbit A. (2016). Host glycosylation pathways and the unfolded protein response contribute to the infection by Francisella. Cell. Microbiol. 18, 1763–1781. 10.1111/cmi.12614 27185209

[B7] Barel M. Meibom K. Dubail I. Botella J. Charbit A. (2012). *Francisella tularensis* regulates the expression of the amino acid transporter SLC1A5 in infected THP-1 human monocytes. Cell. Microbiol. 14, 1769–1783. 10.1111/j.1462-5822.2012.01837.x 22804921

[B8] Bastos P. A. D. da Costa J. P. Vitorino R. (2017). A glimpse into the modulation of post-translational modifications of human-colonizing bacteria. J. Proteomics 152, 254–275. 10.1016/j.jprot.2016.11.005 27888141

[B9] Belyi Y. Tabakova I. Stahl M. Aktories K. (2008). Lgt: a family of cytotoxic glucosyltransferases produced by *Legionella pneumophila*. J. Bacteriol. 190, 3026–3035. 10.1128/JB.01798-07 18281405 PMC2293231

[B10] Brotcke A. Weiss D. S. Kim C. C. Chain P. Malfatti S. Garcia E.. (2006). Identification of Mgla-regulated genes reveals novel virulence factors in *Francisella tularensis*. Infect. Immun. 74, 6642–6655. 10.1128/IAI.01250-06 17000729 PMC1698089

[B11] Case E. D. R. Chong A. Wehrly T. D. Hansen B. Child R. Hwang S.. (2014). The Francisella O-antigen mediates survival in the macrophage cytosol via autophagy avoidance. Cell. Microbiol. 16, 862–877. 10.1111/cmi.12246 24286610 PMC4028363

[B12] Chacko B. K. Scott D. W. Chandler R. T. Patel R. P. (2011). Endothelial surface N-glycans mediate monocyte adhesion and are targets for anti-inflammatory effects of peroxisome proliferator-activated receptor γ ligands. J. Biol. Chem. 286, 38738–38747. 10.1074/jbc.M111.247981 21911496 PMC3207389

[B13] Clemens D. L. Lee B. Y. Horwitz M. A. (2005). *Francisella tularensis* enters macrophages via a novel process involving pseudopod loops. Infect. Immun. 73, 5892–5902. 10.1128/IAI.73.9.5892-5902.2005 16113308 PMC1231130

[B14] Console L. Scalise M. Tarmakova Z. Coe I. R. Indiveri C. (2015). N-linked glycosylation of human SLC1A5 (ASCT2) transporter is critical for trafficking to membrane. Biochim. Biophys. Acta 1853, 1636–1645. 10.1016/j.bbamcr.2015.03.017 25862406

[B15] Dankova V. Balonova L. Link M. Straskova A. Sheshko V. Stulik J. (2016). Inactivation of *Francisella tularensis* gene encoding putative ABC transporter has a pleiotropic effect upon production of various glycoconjugates. J. Proteome Res. 15, 510–524. 10.1021/acs.jproteome.5b00864 26815358

[B16] Dey B. Bishai W. R. (2014). Crosstalk between *Mycobacterium tuberculosis* and the host cell. Semin. Immunol. 26, 486–496. 10.1016/j.smim.2014.09.002 25303934 PMC4250340

[B17] Di Gioia M. Zanoni I. (2015). Toll-like receptor co-receptors as master regulators of the immune response. Mol. Immunol. 63, 143–152. 10.1016/j.molimm.2014.05.008 24951397

[B18] Egge-Jacobsen W. Salomonsson E. N. Aas F. E. Forslund A.-L. Winther-Larsen H. C. Maier J.. (2011). O-linked glycosylation of the pila pilin protein of *Francisella tularensis*: identification of the endogenous protein-targeting oligosaccharyltransferase and characterization of the native oligosaccharide. J. Bacteriol. 193, 5487–5497. 10.1128/JB.00383-11 21804002 PMC3187425

[B19] Hetz C. Martinon F. Rodriguez D. Glimcher L. H. (2011). The unfolded protein response: integrating stress signals through the stress sensor IRE1α. Physiol. Rev. 91, 1219–1243. 10.1152/physrev.00001.2011 22013210

[B20] Lu Q. Li S. Shao F. (2015). Sweet talk: protein glycosylation in bacterial interaction with the host. Trends Microbiol. 23, 630–641. 10.1016/j.tim.2015.07.003 26433695

[B21] Meibom K. L. Charbit A. (2010). The unraveling panoply of *Francisella tularensis* virulence attributes. Curr. Opin. Microbiol. 13, 11–17. 10.1016/j.mib.2009.11.007 20034843

[B22] Miller C. Celli J. (2016). Avoidance and subversion of eukaryotic homeostatic autophagy mechanisms by bacterial pathogens. J. Mol. Biol. 428, 3387–3398. 10.1016/j.jmb.2016.07.007 27456933 PMC5010449

[B23] Moran A. P. Gupta A. Joshi L. (2011). Sweet-talk: role of host glycosylation in bacterial pathogenesis of the gastrointestinal tract. Gut 60, 1412–1425. 10.1136/gut.2010.212704 21228430

[B24] Ni M. Zhou H. Wey S. Baumeister P. Lee A. Y. (2009). Regulation of PERK signaling and leukemic cell survival by a novel cytosolic isoform of the UPR regulator GRP78/BiP. PLoS ONE 4:e6868. 10.1371/journal.pone.0006868 19718440 PMC2729930

[B25] Nizet V. Esko J. (2009). Chapter 39: Bacterial and viral infections, in Essentials of Glycobiology, 2nd Edn., eds Cummings R. D. Varki A. Esko J. D. Freeze H. H. Stanley P. Bertozzi C. R. Hart G. W. Etzler M. E. (Cold Spring Harbor, NY: Cold Spring Harbor Laboratory Press), 1–16. 20301271

[B26] Opdenakker G. Proost P. Van Damme J. (2016). Microbiomic and posttranslational modifications as preludes to autoimmune diseases. Trends Mol. Med. 22, 746–757. 10.1016/j.molmed.2016.07.002 27491925

[B27] Pfaffenbach K. T. Lee A. S. (2011). The critical role of of GRP78 in physiologic and pathologic stress. Curr. Opin. Cell Biol. 23, 150–156. 10.1016/j.ceb.2010.09.007 20970977 PMC3043145

[B28] Ray K. Marteyn B. Sansonetti P. J. Tang C. M. (2009). Life on the inside: the intracellular lifestyle of cytosolic bacteria. Nat. Rev. Microbiol. 7, 333–340. 10.1038/nrmicro2112 19369949

[B29] Santic M. Al Khodor S. Abu Kwaik Y. (2010). Cell biology and molecular ecology of *Francisella tularensis*. Cell. Microbiol. 12, 129–139. 10.1111/j.1462-5822.2009.01400.x 19863554

[B30] Schäffer C. Messner P. (2016). Emerging facets of prokaryotic glycosylation. FEMS Microbiol. Rev. 41, 49–91. 10.1093/femsre/fuw036 27566466 PMC5266552

[B31] Sjöstedt A. (2011). Special topic on *Francisella tularensis* and tularemia. Front. Cell. Infect. Microbiol. 2:86. 10.3389/fmicb.2011.00086 21833327 PMC3153047

[B32] Tan F. Y. Y. Tang C. M. Exley R. M. (2015). Sugar coating: bacterial protein glycosylation and host–microbe interactions. Trends Biochem. Sci. 40, 342–350. 10.1016/j.tibs.2015.03.016 25936979

[B33] Thomas R. M. Twine S. M. Fulton K. M. Tessier L. Kilmury S. L. N. Ding W.. (2011). Glycosylation of DsbA in francisella tularensis subsp. tularensis. J Bacteriol 193, 5498–5509. 10.1128/JB.00438-11 21803994 PMC3187430

[B34] Tytgat H. L. P. de Vos W. M. (2016). Sugar coating the envelope: glycoconjugates for microbe–host crosstalk. Trends Microbiol. 24, 853–861. 10.1016/j.tim.2016.06.004 27374775

[B35] Vaidyanathan K. Durning S. Wells L. (2014). Functional O-GlcNac modifications: implications in molecular regulation and pathophysiology. Crit. Rev. Biochem. Mol. Biol. 49, 140–163. 10.3109/10409238.2014.884535 24524620 PMC4912837

[B36] Varki A. (1993). Biological roles of oligosaccharides: all of the theories are correct. Glycobiology 3, 97–130. 10.1093/glycob/3.2.97 8490246 PMC7108619

[B37] Walters K.-A. Olsufka R. Kuestner R. E. Cho J. H. Li H. Zornetzer G. A.. (2013). *Francisella tularensis* subsp. tularensis induces a unique pulmonary inflammatory response: role of bacterial gene expression in temporal regulation of host defense responses. PLoS ONE 8:e62412. 10.1371/journal.pone.0062412 23690939 PMC3653966

[B38] Zhang Y. J. Rubin E. J. (2013). Feast or famine: the host–pathogen battle over amino acids. Cell. Microbiol. 15, 1079–1087. 10.1111/cmi.12140 23521858 PMC6434321

